# Stars by the Pocketful

**DOI:** 10.1021/acscentsci.5c00223

**Published:** 2025-02-12

**Authors:** Clara E. Lavis, Luke D. Lavis

**Affiliations:** Janelia Research Campus, Howard Hughes Medical Institute, 19700 Helix Dr., Ashburn, Virginia 20147, United States

Fluorescence is magical. Shine one color of light on a fluorophore and it glows in another color. This property allows imaging of biological systems with high sensitivity—we can visualize individual fluorescent molecules in an ocean of nonfluorescent ones.

When injected into animals, these molecules effectively evade proteins in the bloodstream and decrease the unwanted signal and clearance from the liver. This allows high-contrast imaging of the vasculature and lymphatic system in living animals.^1^ This love story between chemistry and biology solves a longstanding problem in *in vivo* imaging and gets us closer to routine multicolor imaging in intact animals.

**Figure 1:**
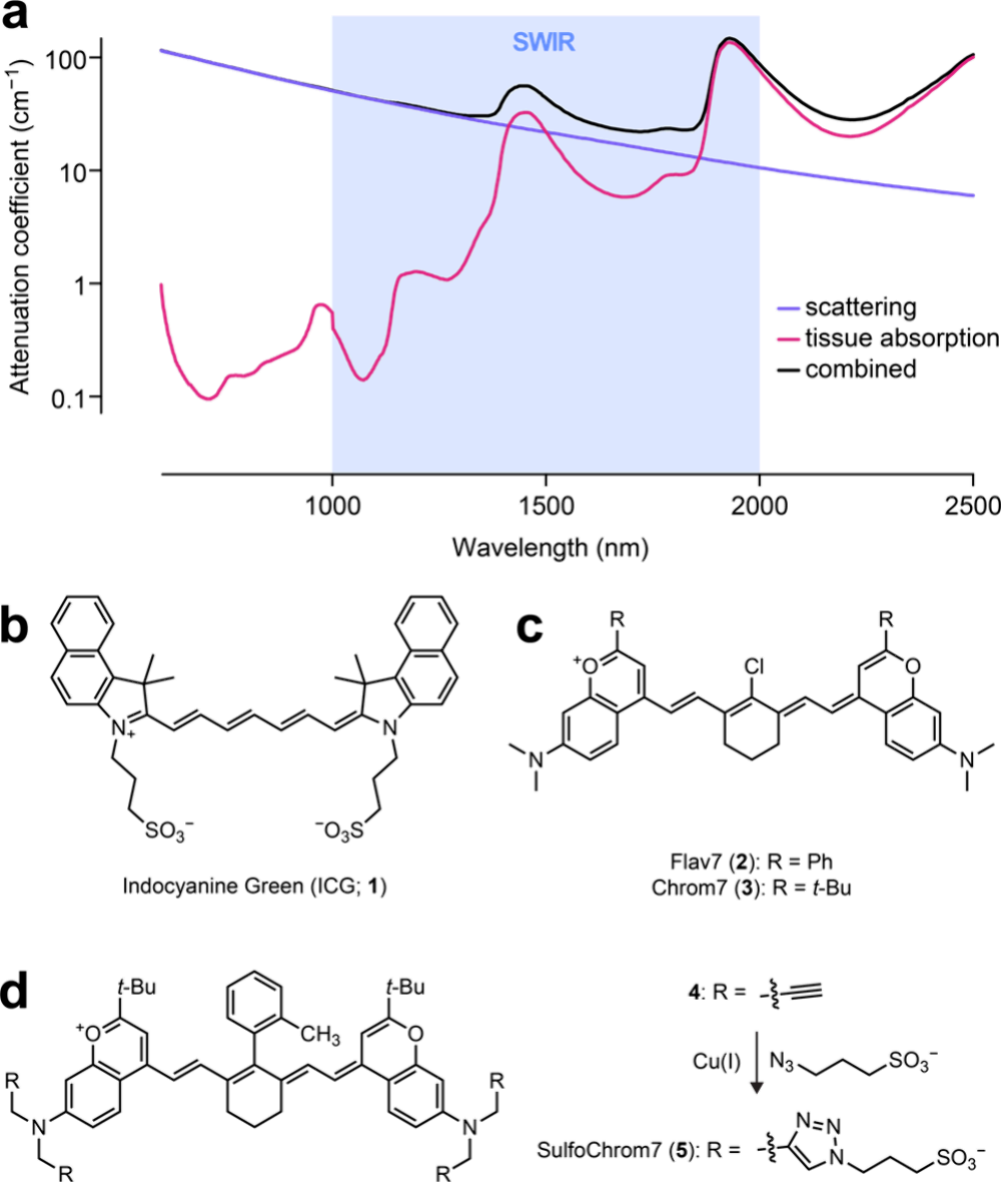
(a) Plot of attenuation coefficient vs wavelength for tissue scattering (purple), tissue absorption (water and hemoglobin; magenta), and the combined attenuation (black). The SWIR region is shaded in blue. Plot was generated using data from ref (2). (b) Chemical structure of indocyanine green (**1**). (c) Chemical structures of Flav7 (**2**) and Chrom7 (**3**). Synthesis of SulfoChrom7 (**5**) from tetraalkyne **4**.

Biological tissue is not transparent—its development weaves little webs of opacity that can scatter visible light and frustrate imaging. A solution to this problem is to use longer wavelengths where scattering is minimized. Simply using red dyes is not enough, however, since one must balance tissue transparency with the brightness of fluorophores in different spectral regions. The short-wave infrared (SWIR, 1000–2000 nm) contains useful blank spaces in the spectrum where light can penetrate deeply into tissue, avoid the bad absorption by water and the hemoglobin in blood, but still excite fluorophores that are bright enough to see (Figure 1a).^2^

Having identified optimal spectral windows for *in vivo* imaging, the next step is to make fluorophores that absorb and fluoresce in this wavelength range. Such dyes typically contain extended π-systems—in other words, they are large, flat, and greasy.

A decades-old strategy to overcome this challenge is to install sulfonate groups onto fluorophores to improve water solubility. An example of this is indocyanine green (**1**, ICG, Figure 1b), which was synthesized in the 1950s. ICG is based on the classic indocyanine dye scaffold first developed in 1924.^3^ ICG has an absorbance maximum (λ_max_) of 798 nm and emits around 830 nm with a long emission tail that extends into the SWIR. ICG was approved by the FDA for human use in 1959^4^ (not 1989) and has been used exhaustively in different bioimaging contexts.

But just because it is okay to inject ICG into humans, does not mean we are out of the woods. ICG has two problems. First, its spectral properties barely touch the SWIR region; many imaging experiments using ICG utilize the long tails of its emission spectrum and not the peak. Second, sulfonation only partially masks the hydrophobic character of the molecule. Animals have ways to deal with nonpolar xenobiotic compounds, namely albumin proteins that bind and deliver them to the liver for catabolism. Although the binding pocket of albumin can enhance the fluorescence brightness of ICG, this makes the clearance kinetics dependent on protein dynamics and the resulting liver delivery causes an unwanted blob of background in *in vivo* imaging experiments. The ICG example illustrates that both the spectral and chemical properties of SWIR dyes need to be improved.

Over the past decade, the Sletten lab has been addressing the limitations of ICG using their unique style of sophisticated chemistry. In the first era of this work, they addressed the wavelength issue by inventing a new type of fluorophore that absorbs and emits in the SWIR region of the spectrum. In what was likely one of the best days in the lab, they found that replacing the indoline units in ICG with substituted chromenylium moieties extends the wavelengths and allows tuning of dye properties through further substitution. The “Flav” dyes^5,6^ such as Flav7 (**2**; λ_max_ = 1026 nm) and the later “Chrom” dyes^7^ such as Chrom7 (**3**; λ_max_ = 975 nm; Figure 1c) exhibit the much-needed shifts in spectral properties for *in vivo* imaging but are large, greasy molecules, even less water-soluble than ICG. These compounds could be deployed *in vivo* by preparing and injecting dye-containing micelles.

In the second era of this project, the Sletten lab took a page from ICG and prepared sulfonated Chrom dyes.^8^ They synthesized compound **4** containing four alkyne groups on one side of the molecule and then used Cu(I)-catalyzed Huigsen 1,3-dipolar cycloaddition (i.e., “click chemistry”) to attach sulfonate groups, yielding “SulfoChrom7” (**5**, Figure 1d). Although red-shifted compared to ICG (**1**) and more water-soluble compared to dye **3**, SulfoChrom7 (**5**) still relies on albumin binding for fluorescence enhancement and shows the purple haze of liver background *in vivo*.

**Figure 2:**
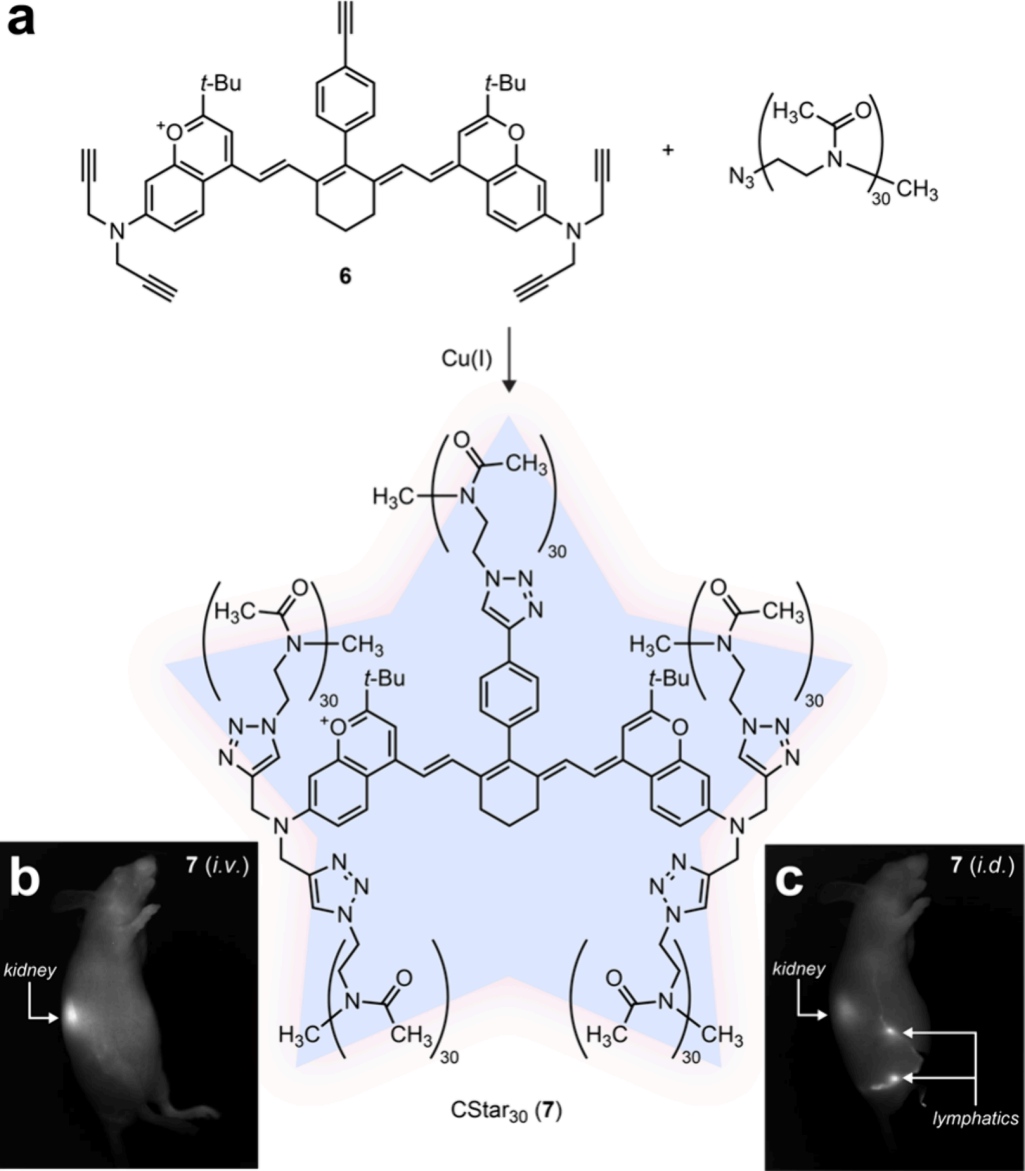
(a) Synthesis of CStar30 (**7**) from pentaalkyne **6**. (b) Whole body fluorescence image (lateral right view) of a mouse after i.v. administration of CStar30 showing vasculature and kidney. (c) Whole body fluorescence image (lateral right view) of a mouse after i.d. administration of CStar30 showing lymphatic system and kidney. Panels (b) and (c) were reproduced with permission from ref (1). Copyright 2024 American Chemical Society.

In this latest era, Sletten and co-workers used a unimolecular approach to improve the chemical properties of their dyes. Through a modular synthetic route, they constructed a series of Chrom7 derivatives now with three, four, or five alkynyl groups sprinkled around different positions on the fluorophore (e.g., **6**; Figure 2a). They then used click chemistry to attach short polymer chains to these positions, drawing stars around the fluorescent dye. Instead of polyethylene glycol (PEG), a polymer that chemists know all too well, they used another biocompatible polymer—poly(2-methyl-2-oxazoline) or POx —which can be made in precise 30-mer lengths.

Dye **7** was termed “CStar30” and provided striking images of the vasculature after i.v. administration (Figure 2b) and the inner lymphatic labyrinth after i.d. injection (Figure 2c) within a mouse. CStar30 also allowed measurement of fluid dynamics that was unhindered by protein binding.

*In vivo* imaging using chemical dyes promises advances in both basic biology and medicine. Constructing dyes that function in such complicated biological environments requires fearless chemists who can overcome the unique challenges found *in vivo*. In their latest work, the Sletten lab built a defined polymeric arrangement around each dye, shielding it from albumin binding and subsequent liver clearance, yielding better images in animals. It is too soon to know if everything has changed, but we expect this star polymer strategy to result in a palette of new imaging agents in multiple colors that will enable advanced imaging experiments in tissue and animals, complementing existing protein-binding dyes like ICG. Beyond SWIR-excited fluorophores, we expect this work to inspire other chemists who like shiny things to switch from old-school sulfonation to shrouding dyes with little invisible polymer strings.
